# Erythrasma: Pathogenesis and Diagnostic Challenges

**DOI:** 10.7759/cureus.68308

**Published:** 2024-08-31

**Authors:** Jesús Iván Martínez-Ortega, Samantha Franco González

**Affiliations:** 1 Dermatology, Dermatology Institute of Jalisco, Zapopan, MEX; 2 Histology, Autonomous University of Nuevo Leon, Monterrey, MEX; 3 Internal Medicine, National Medical Center Century XXI, Ciudad de México, MEX

**Keywords:** differential diagnosis, pathogenesis, uv led light, ultraviolet-induced fluorescence dermoscopy, coproporphyrin iii, wood's lamp, diagnostic techniques, porphyrins, corynebacterium minutissimum, erythrasma

## Abstract

Erythrasma is a common chronic superficial bacterial infection caused by *Corynebacterium minutissimum*, a lipophilic, diphtheroid, filamentous, gram-positive bacillus and normal inhabitant of the skin flora. Commonly found in intertriginous spaces, this bacterium releases a porphyrin that causes lesions to fluoresce under a Wood’s lamp, aiding diagnosis. Despite its clinical relevance, the pathogenesis remains understudied. We present a case of a 43-year-old woman with a history of hypertension, type 2 diabetes mellitus, and obesity who exhibited an erythematous eczematous plaque with characteristic coral-red fluorescence under Wood’s lamp. The diagnosis was confirmed through negative skin scrapings for candidiasis and dermatophytosis. A two-week course of topical clindamycin resulted in complete resolution. Given the condition’s frequent coexistence with other pathologies, accurate diagnosis and effective treatment are essential. Topical treatments are usually more convenient. The hypothesis that hydrophobic and lipophilic interactions in intertriginous areas contribute to the pathogenesis of erythrasma requires further experimental validation.

## Introduction

Erythrasma is a common chronic superficial bacterial infection caused by *Corynebacterium minutissimum*, a gram-positive, lipophilic, filamentous bacillus that is a normal component of the skin flora. This diphtheroid bacterium typically colonizes areas such as the genital regions, interdigital spaces of the feet, and intertriginous zones, often in conjunction with other microorganisms. The infection frequently occurs in individuals with specific risk factors such as diabetes, obesity, and poor hygiene, particularly in warm and humid environments, which create conditions that favor the proliferation of the bacteria. Clinically, patients are usually asymptomatic or present with mild pruritus, erythema, maceration, scaling, or skin discoloration, and in many cases, erythrasma may be incidentally discovered during examination for other conditions. *C. minutissimum *produces a porphyrin pigment that causes lesions to exhibit a characteristic coral-red fluorescence under a Wood’s lamp, a diagnostic tool that effectively facilitates accurate diagnosis and serves as a surrogate for laboratory testing [1,2]. This case highlights the importance of recognizing these risk factors and utilizing the distinctive coral-red fluorescence for diagnosis, leading to effective topical treatment.

## Case presentation

**Figure 1:**
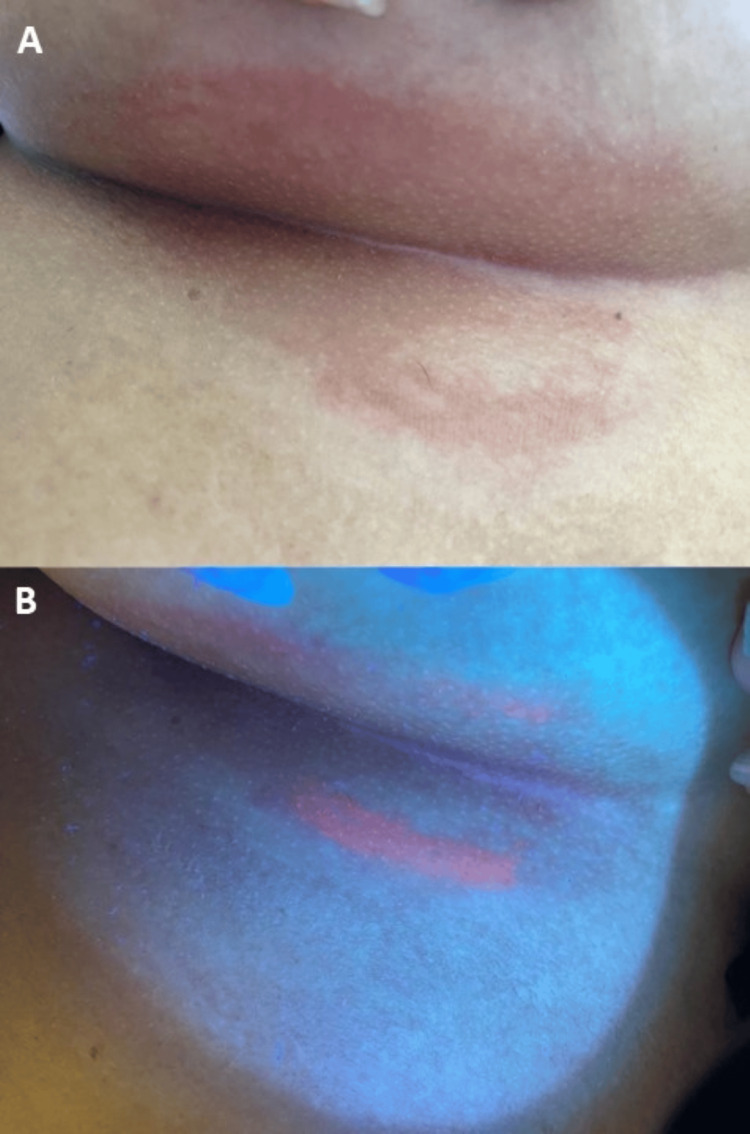
Clinical presentation and diagnosis of erythrasma Panel A: Physical examination under normal light. Panel B: Wood’s lamp examination of the same lesion, revealing characteristic coral-red fluorescence at the lesion’s periphery, indicative of erythrasma. Direct examination of skin scrapings was performed to rule out candidiasis and dermatophytosis, and the results were negative.

A 43-year-old woman with a history of hypertension, type 2 diabetes mellitus, and obesity presented with itching and redness for 15 days. Physical examination revealed an erythematous eczematous plaque in the right inframammary fold. Wood’s lamp examination showed a coral-red fluorescence at the lesion’s periphery. To rule out candidiasis and dermatophytosis, direct examination of skin scrapings was performed using KOH preparation and wet mount preparation, with negative results for dermatophytes and candidiasis (Figure 1). There was no history of the recent introduction of new chemicals, such as deodorants, skin-care products, or the use of harsh soaps with abrasive scrubs. Since the Wood’s lamp test confirmed the diagnosis of erythrasma, further cultures were deemed unnecessary. A two-week course of topical clindamycin was prescribed, leading to complete resolution of the condition, which was confirmed at the one-month follow-up.

## Discussion

The condition was first described in 1859 by Burchardt, who initially attributed it to a fungal cause [2-4]. However, in 1862, the German dermatopathologist Friedrich Wilhelm Felix von Bärensprung introduced the term “erythrasma” and initially identified the causative organism as *Microsporum minutissimum* [2]. This bacterium was later reclassified as *C. minutissimum* [2-4].

There is a lack of epidemiologic data on erythrasma, and the reported prevalence varies greatly from one study to another. Some have reported a prevalence of 0.47% [5] and others 14% [6] and 33% [1]. Population heterogeneity and other variables such as diagnosis method, geology topography, and selection bias may influence the variability.

Additionally, certain groups may have higher incidence and prevalence. Erythrasma was identified in 39% of athletes and 40% of soccer players [7]. A study of military recruits reported a prevalence of 77% [8]. The reported prevalence in businessmen is 50% and in housewives is 37.5%; additionally, the prevalence of erythrasma in patients with diabetes is 50% and 62.5% among those who are obese [3]. These increased rates can be attributed to various factors. For example, the humidity associated with certain professions or occupations, such as athletes and military recruits, contributes to sweat accumulation and skin maceration, which can promote the growth of *C. minutissimum*. Additionally, conditions like obesity and diabetes are linked with local humidity due to maceration in skin folds and reduced immune function, respectively. Our case highlights the classic risk factors for erythrasma, including the patient’s history of diabetes and obesity, which are well-established contributors to the development of this infection. These factors, combined with the warm and moist environment of the inframammary fold, likely facilitated the transition from a commensal state to a pathogenic one [2].

Taxonomically, the genus *Corynebacterium* includes species such as *C. minutissimum*, which causes erythrasma; *C. flavescens*, previously known as *C. tenuis*, is responsible for trichobacteriosis (trichomycosis axillaris) in its flava variant. *Kytococcus sedentarius*, a distinct genus, is primarily implicated in pitted keratolysis. Additionally, other bacteria, including certain *Corynebacterium* species, *Actinomyces* species, and *Dermatophilus* species, have also been associated with pitted keratolysis [2,3].

Erythrasma does not result from exogenous contamination but rather from local environmental changes, such as warm and moist conditions under occlusion, which favor the shift from commensalism to infection. Despite its clinical relevance, the pathogenesis of this infection remains understudied and scarce in the literature [1]. In 1884, Köbner established that erythrasma could be transmitted by transferring epidermal scales from an infected person to a healthy one [2]. Subsequently, in 1961, the bacterium responsible for erythrasma was isolated from clinical lesions for the first time, thereby validating its role in the disease [2,9].

*C. minutissimum* is a lipophilic bacterium that produces coproporphyrin III, a porphyrin [1]. While the role of this compound in the pathophysiology of erythrasma remains unclear, its clinical relevance lies in its ability to fluoresce coral-red under UV light. The UV light has a peak emission at 365 nm, which is absorbed by coproporphyrin III, resulting in fluorescence in the red spectrum at around 620 nm [10,11].

Although *C. minutissimum* has been reported to cause keratolysis in superficial skin lesions, clinical observations suggest that the extent of keratolysis is less severe compared to that caused by dermatophytes. It is also possible that the aggregation and infectivity of lipophilic bacteria like *C. minutissimum* in intertriginous areas can be explained by similar principles to those observed in hydrophobic fungi. The hydrophobic lipid matrix of the stratum corneum creates an environment conducive to hydrophobic interactions, which facilitate the aggregation and stability of hydrophobic and lipophilic organisms [12].

In the case of *C. minutissimum*, the presence of warm, moist, and saline conditions in intertriginous areas may promote the clumping together of these lipophilic bacteria. This aggregation would reduce their exposure to water and increase their stability within the lipid-rich environment of the stratum corneum, much like how hydrophobic fungi would aggregate to minimize water exposure [12].

Additionally, the accumulation of *C. minutissimum* in these areas may cause an overload of pattern recognition receptors (PRRs) activation due to interactions with bacterial components or cellular products from keratolysis in keratinocytes. This can lead to sustained inflammatory responses. Specifically, the mycolic acids present on the bacterial surface have been shown to activate IL-17/IL-23 signaling pathways in the skin [13,14]. Furthermore, the porphyrins produced by *C. minutissimum* can trigger activation of the NLRP3 inflammasome in keratinocytes, contributing to the inflammatory process. This activation may contribute to an inflammatory environment marked by the release of cytokines and other inflammatory mediators [13], ultimately leading to the clinical manifestations of erythrasma.

**Figure 2:**
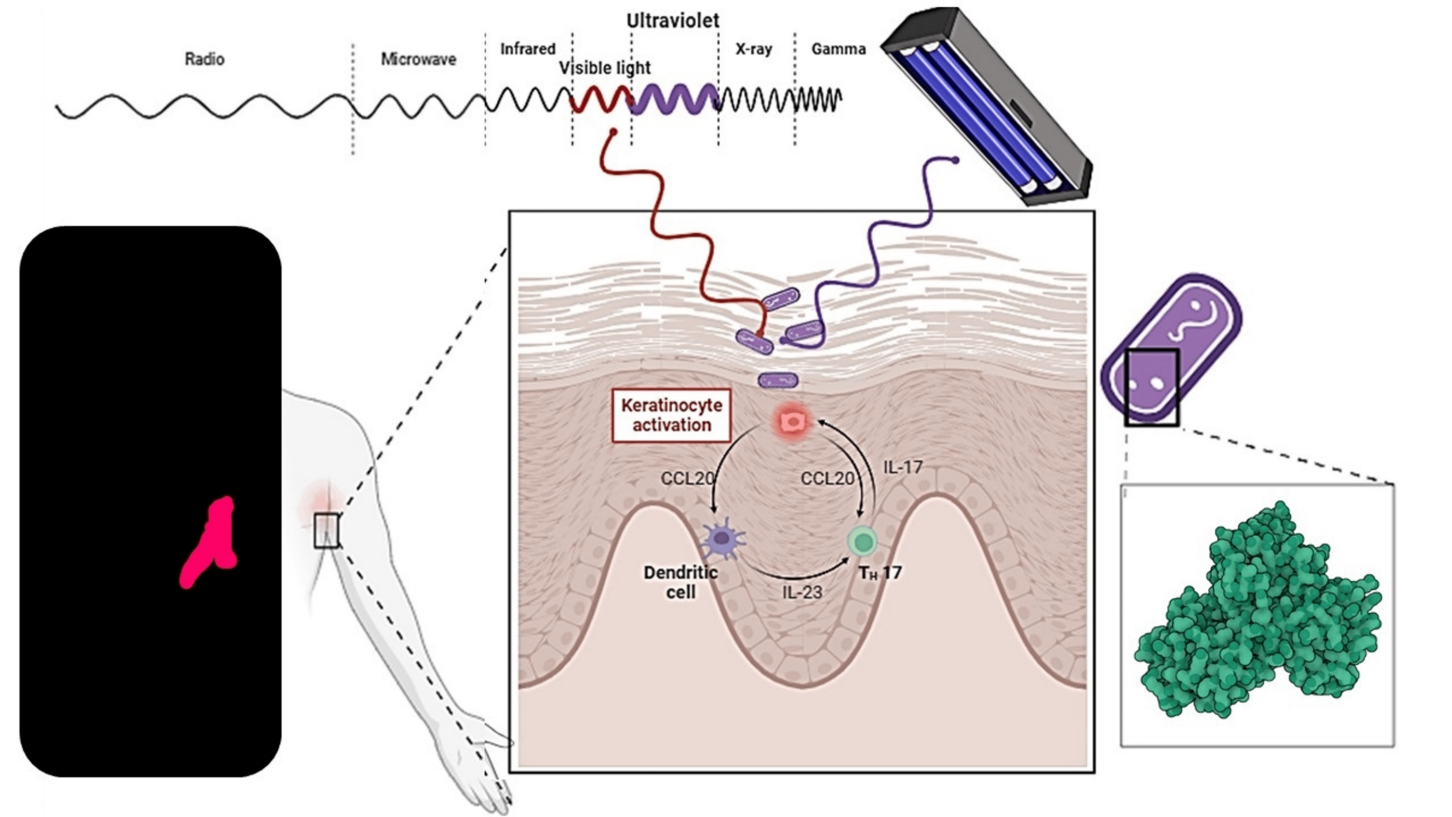
Pathophysiology of erythrasma The combined figure is created with BioRender.com and the design of coproporphyrin III (in green) by AlphaFold Protein Structure Database (https://alphafold.ebi.ac.uk), depicting the hypothesis that the aggregation of lipophilic bacteria in intertriginous areas is facilitated by hydrophobic interactions within the stratum corneum, which are favored in a humid medium. This promotes the transition from commensalism to infection and inflammatory responses. Coproporphyrin III is proposed to play a dual role: contributing to the inflammatory response and causing the coral-red fluorescence under UV light. Specifically, UV light has a peak emission at 365 nm, which is absorbed by coproporphyrin III, leading to fluorescence in the red spectrum at around 620 nm. This UV-based fluorescence acts as a biomarker for activity and may serve as a tool for distinguishing between commensalism and infection. Image credits: Jesus Martinez MD.

In summary, we hypothesize that hydrophobic and lipophilic interactions contribute to the aggregation and increased infectivity of *C. minutissimum* in saline and humid intertriginous areas, leading to an inflammatory response and the clinical symptoms of erythrasma. Experimental studies are needed to validate this hypothesis. Given that *C. minutissimum* is a commensal organism, direct examination may not effectively differentiate between commensalism and active infection, which could explain its frequent coexistence with other conditions like candidiasis or inverse psoriasis [1,15]. If porphyrins are shown to have a direct correlation with inflammation, morphometric analysis could establish a threshold for coloration signals, allowing for a more reliable diagnosis of infection (Figure 2). It is crucial to note that these proposed mechanisms are speculative and have not yet been validated by experimental studies. Further research is needed to confirm these hypotheses.

Most patients with erythrasma are asymptomatic or with minimal symptoms such as mild pruritus or skin discoloration; indeed, in many patients, erythrasma may be discovered by chance while examining the patient for other conditions, but most of the patients present erythema, maceration, and scaling [3,4].

Erythrasma commonly affects areas prone to moisture and friction, such as the axilla, submammary fold, groin, intergluteal region, periumbilical area, perianal area, and between the toes. These regions are typically characterized by conditions that facilitate moisture retention and friction, contributing to the development of erythrasma [1,2,4].

The differential diagnosis of erythrasma is crucial, yet no established guidelines currently exist. To address this gap, we have created a table with what we consider the most common differential diagnoses (Table 1) [1,2,15-18]. Notably, for about half of these conditions, the Wood’s lamp offers a practical and useful diagnostic tool. Although its sensitivity and specificity have not been firmly established, it is considered a valuable initial method for evaluating mild flexural fold symptoms. In the absence of a Wood’s lamp, using a UV LED light from a smartphone or other sources has been suggested as an alternative [19].

**Table 1:** Differential diagnosis of erythrasma This table outlines the differential diagnoses for erythrasma, including key diagnostic features, and notes which conditions may present positive results under specific diagnostic methods. Note that half of the differential diagnoses benefit from UV lamps; also, note that only erythrasma produce a coral red; finally, although most of the diagnoses do not require biopsy, in cases of no response or uncertainty, it may be indicated and may be beneficial, as shown in the right column. *Tinea versicolor is a recently proposed term for pityriasis versicolor affecting intertrigo areas. The information in this table is based on data from references [1], [2], and [12-15]. The differential diagnosis table includes common conditions based on our clinical experience but does not cover every possible diagnosis. The insights reflect our observations and aim to provide a practical overview within the context of the described case. The table should be interpreted as a guide rather than an exhaustive review of all potential conditions. Table credits: Jesus Martinez MD.

Condition	Positive to Wood's lamp/UV light	Positive to direct exam	Biopsy indicated	Main findings
Erythrasma	Yes (coral-red fluorescence)	Yes (gram-positive rods on microscopy)	No	Coral-red fluorescence under Wood's lamp; gram-positive rods on direct microscopy, although Wood's lamp examination is more definitive for diagnosis.
Tinea infections (e.g., pedis, axillaris, etc.)	Yes (e.g., *Microsporum canis* shows blue-green fluorescence)	Yes (KOH prep shows hyphae and spores)	No	KOH prep shows hyphae and spores; *Microsporum canis* produces blue-green fluorescence under Wood's lamp.
*Candida albicans* infection	No	Yes (wet mount preparation with saline solution shows budding yeast and pseudohyphae)	No	Wet mount preparation reveals budding yeast and pseudohyphae; it typically affects moist areas.
*Malassezia furfur* (pityriasis versicolor/tinea versicolor)*	Yes (yellow-orange fluorescence)	Yes (methylene blue or KOH prep shows hyphae and "spaghetti and meatballs" appearance)	No	KOH/methylene blue prep shows hyphae and yeast; yellow-orange fluorescence under Wood's lamp.
Gram-negative toe web infections	Yes (green fluorescence if caused by *Pseudomonas*)	No	No	Biopsy may reveal Gram-negative rods; *Pseudomonas* produces green fluorescence under Wood's lamp.
Post-inflammatory pigmentation	No	No	No	Hyperpigmented areas resulting from previous inflammation; direct examination and Wood's lamp are negative. Although a biopsy may reveal melanophages in the dermis, it is usually not necessary, as a thorough clinical evaluation is typically sufficient for diagnosis.
Intertrigo	No	No	No	Inflammatory reaction in skin folds, often due to friction and moisture; no specific findings on microscopy. Similar to hyperpigmentation, a thorough clinical evaluation is usually sufficient for diagnosis, as biopsy and other tests are generally not required.
Inverse psoriasis	No	No	Yes	Biopsy shows hyperkeratosis (typically parakeratosis), acanthosis, and Munro microabscesses; KOH prep is negative. Although the clinical presentation often suggests the diagnosis, biopsy is necessary to confirm.
Contact dermatitis	No	No	No	Biopsy reveals spongiosis, vesiculation, and inflammation; KOH prep is negative. Although clinical diagnosis is usually sufficient, a patch test may be more useful than a biopsy, which is not typically performed.
Seborrheic dermatitis	No	No	No	Biopsy shows parakeratosis and inflammation in the upper dermis; KOH prep is negative. Although the condition may be associated with Malassezia, direct examination for Malassezia is not necessary. Clinical evaluation is usually sufficient for diagnosis. Parakeratosis in seborrheic dermatitis typically lacks the Munro microabscesses seen in psoriasis, helping to differentiate between the two conditions.

Additionally, the application of dermoscopy may enhance the accuracy and utility of the Wood’s lamp, although its efficacy is still under evaluation and holds high expectations [20]. Given reports of erythrasma coexisting with other pathologies, we recommend screening all patients with suspected symptoms for erythrasma using Wood’s lamps, UV LED lights, or ultraviolet-induced fluorescence dermoscopy.

Direct examination of suspected cases can be performed using various methods. Wet mount preparation is frequently used for diagnosing candidiasis, where a sample from skin, nails, or mucous membranes is placed on a slide with a drop of KOH solution. The KOH dissolves keratin and other cellular material, making it easier to visualize yeast cells or pseudohyphae under a microscope. Methylene blue staining involves mounting scale samples on a slide or using transparent adhesive tape and then staining for 2-3 minutes to highlight fungal elements. Gram staining reveals rods (either isolated or in chains) and intertwining filaments measuring between 4 and 7 μm, along with coccoid forms ranging from 1 to 3 μm. This method shows thin, straight, or slightly curved gram-positive bacilli with club-shaped ends, forming a distinctive V-shaped or Chinese letter (cuneiform) pattern. Culturing is generally challenging and typically unnecessary for diagnosis [1,4]. 

There is no consensus on the treatment of erythrasma, and there are no established guidelines or extensive real-world studies to guide treatment decisions. However, local treatment is often recommended for mild, localized infections. In practice, most cases are indeed mild and localized, so topical treatment is usually sufficient. Topical therapies, such as fusidic acid cream (2%), mupirocin (2%), or clindamycin (1%), are preferred due to their lower risk of adverse effects and better patient adherence [1,2,21]. Topical treatments, notably fusidic acid cream, are effective and have superior patient compliance compared to systemic therapies. For instance, in some studies, fusidic acid cream has been found to be more effective than oral clarithromycin and erythromycin. The typical duration of topical treatment is seven to 14 days, applied twice daily [21].

For more extensive or resistant cases, oral treatment may be necessary. Oral antibiotics, such as erythromycin (250 mg four times daily) or clarithromycin (500 mg twice daily), are commonly used, with a typical treatment duration of seven to 10 days. The decision to use oral antibiotics is generally based on the extent of the infection, the patient’s immune status, and the response to initial topical therapy [1,4,21].

## Conclusions

Erythrasma, caused by *C. minutissimum*, is a frequently overlooked superficial bacterial infection that predominantly affects intertriginous areas. Diagnosis is facilitated by the distinctive coral-red fluorescence under a Wood’s lamp, although alternative methods such as UV LED lights and UV-dermoscopy may also be useful. This case underscores the importance of considering erythrasma in differential diagnoses, particularly in patients with comorbid conditions like diabetes and obesity. Topical treatments, notably fusidic acid cream, are effective and have superior patient compliance compared to systemic therapies. Understanding the role of hydrophobic and lipophilic interactions and the involvement of porphyrins in the pathogenesis of erythrasma could enhance diagnostic accuracy and allow better strategies.
